# Liquid-liquid phase separation: a new perspective on respiratory diseases

**DOI:** 10.3389/fimmu.2024.1444253

**Published:** 2024-09-26

**Authors:** Li Wang, Yongjun Wang, Zhangmin Ke, Zexu Wang, Yufang Guo, Yunlei Zhang, Xiuwei Zhang, Zhongliang Guo, Bing Wan

**Affiliations:** ^1^ Department of Respiratory and Critical Care Medicine, The Affiliated Jiangning Hospital of Nanjing Medical University, Nanjing, China; ^2^ Shanghai East Clinical Medical College, Nanjing Medical University, Nanjing, China

**Keywords:** liquid-liquid phase separation, membrane-less organelle, biomolecular condensates, respiratory diseases, lung

## Abstract

Liquid-liquid phase separation (LLPS) is integral to various biological processes, facilitating signal transduction by creating a condensed, membrane-less environment that plays crucial roles in diverse physiological and pathological processes. Recent evidence has underscored the significance of LLPS in human health and disease. However, its implications in respiratory diseases remain poorly understood. This review explores current insights into the mechanisms and biological roles of LLPS, focusing particularly on its relevance to respiratory diseases, aiming to deepen our understanding and propose a new paradigm for studying phase separation in this context.

## 1 Introduction

**Figure 1:**
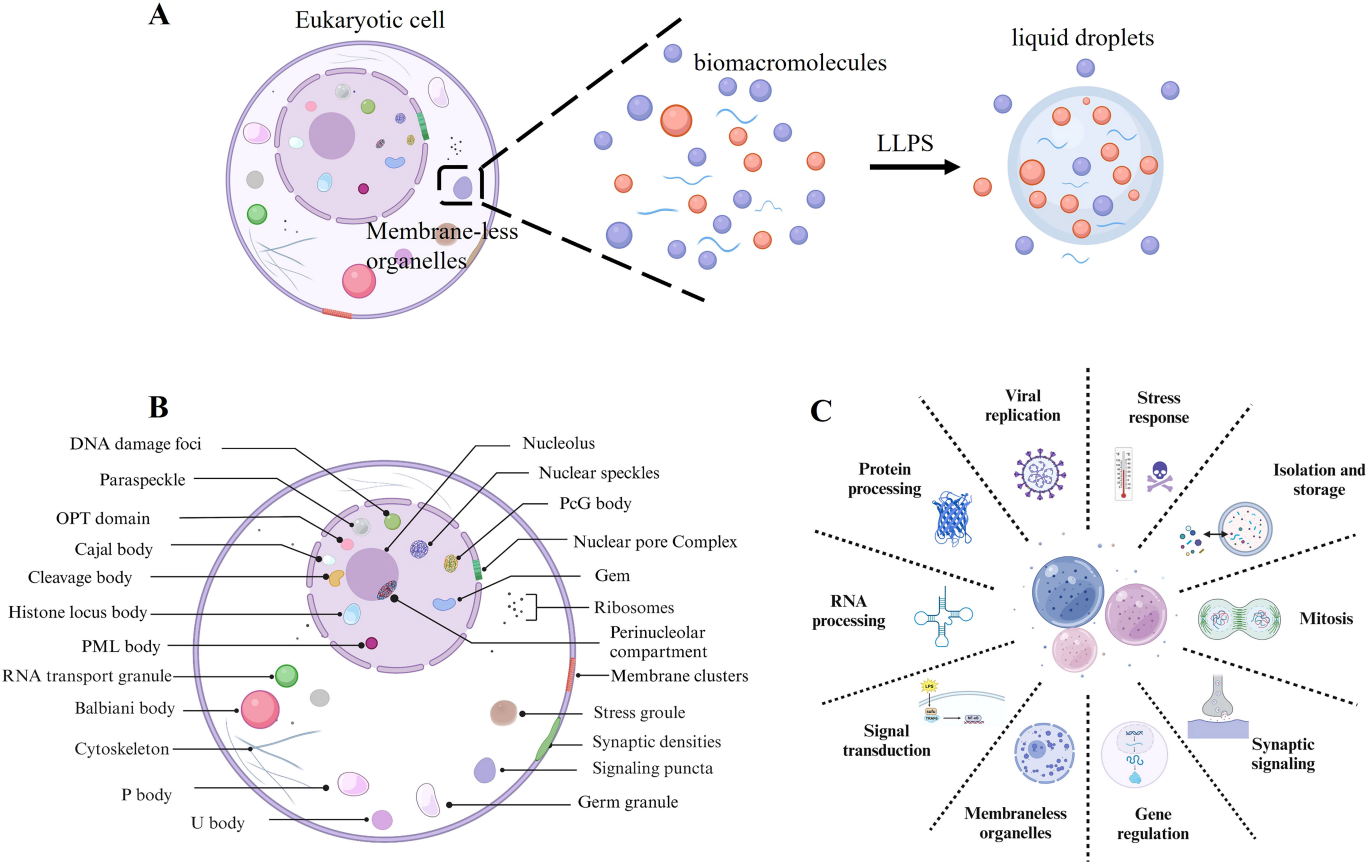
Liquid-liquid phase separation. **(A)** Biomacromolecules undergo LLPS to form liquid droplets, which contributes to the formation of membrane-less organelles. **(B)** Examples of membrane-less organelles include those found distributed throughout the nucleus, nuclear membrane, cytoplasm, and plasma membranes of eukaryotic cells. **(C)** Physiological function of LLPS.

Liquid-liquid phase separation (LLPS) is a fundamental biophysical phenomenon in cellular biology where biomolecules, such as proteins and RNA, spontaneously aggregate into distinct regions within the cytoplasm or nucleus. These regions exhibit unique physical and chemical properties compared to their surroundings (1, 2). Analogous to the separation of oil and water, LLPS results in the formation of two distinct liquid phases. Facilitated by dynamic and reversible interactions among proteins and nucleic acids, LLPS enables the creation of membrane-less intracellular compartments, giving rise to organelles that lack traditional lipid bilayers (**Figure 1A**) (3, 4). These structures are crucial for organizing cellular components spatially, thereby regulating diverse cellular functions effectively (5).

LLPS facilitates the formation of membrane-less organelles such as nucleoli, stress granules (SGs), Cajal bodies, and P-bodies. These structures compartmentalize cellular components without lipid bilayers, thereby establishing specialized biochemical environments (5–7) (**Figure 1B**). This spatial segregation markedly enhances the efficiency and precision of biochemical reactions within the complex cellular milieu (8–10). Moreover, LLPS is crucial for cellular organization and regulation, affecting a variety of essential cellular processes (**Figure 1C**). Firstly, LLPS regulates gene expression and RNA processing by forming transcriptional hubs that localize transcription factors and regulatory molecules near specific genes (11, 12). This localization can modulate gene expression, either enhancing or repressing it as needed. In RNA processing and translation, LLPS facilitates the formation and regulation of ribonucleoprotein complexes essential for RNA splicing, transport, stabilization, and translation regulation (13). Similarly, in signal transduction and stress response, LLPS assumes distinctive roles (13). It modulates signal transduction pathways by localizing signaling molecules within phase-separated domains, which enhances or diminishes signal transmission (14). This concentration of signaling molecules enables efficient signal integration and modulation within the cell. During stress conditions, cells utilize LLPS to create SGs that sequester and protect RNA and proteins vital for cellular survival in hostile environments. For instance, in response to heat shock or oxidative stress, cells form SGs via LLPS to shield essential molecular components (15). Furthermore, LLPS is instrumental in protein sequestration and degradation. It aids in creating proteasome-rich compartments for targeted protein degradation and serves as a mechanism for protein storage (16). LLPS has been implicated in controlling cell cycle regulation. Phase-separated compartments concentrate cell cycle regulators, ensuring orderly progression through the cell cycle phases. Beyond these functions, LLPS regulates the spatial and temporal regulation of proteins and RNAs, which are vital for cell differentiation and organ development (17). Lastly, the dynamic nature of LLPS, characterized by the formation and resolution of compartments in response to cellular needs, underscores its essential role in maintaining cellular homeostasis and adapting to environmental changes (18).

**Figure 2:**
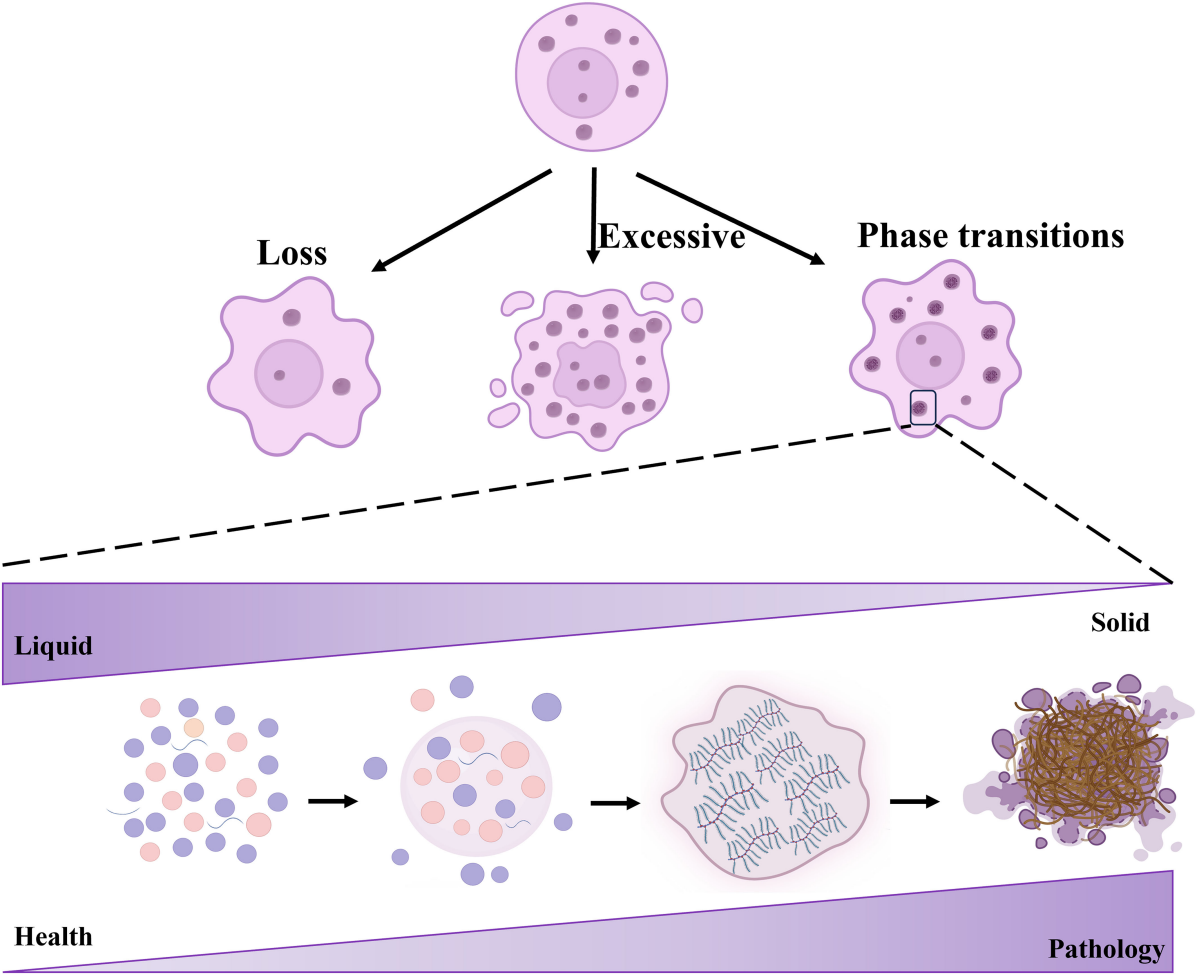
Aberrant LLPS encompasses loss, excessive condensation, and abnormal phase transitions. Biomacromolecules undergo LLPS to form droplets, initially reversible but over time transitioning to more solid-like states such as fibers or hydrogels.

Notably, the dysregulation of LLPS is associated with various diseases, such as cancer and neurodegenerative disorders (5, 19–21). Aberrant LLPS in cellular contexts manifests in several forms, including loss, excessive condensation, and abnormal phase transitions, all of which significantly impact cellular organization and function (**Figure 2**). Beginning with the loss of LLPS, a decrease in this process disrupts the normal formation of membrane-less organelles such as nucleoli, SGs, and P-bodies (22). This deficiency results in the mislocalization of proteins and RNA, undermining essential cellular functions, particularly notable in diseases such as muscular dystrophies and certain cancers, where RNA-binding proteins affected by LLPS loss play a critical role (8, 14, 23). For instance, mutations impairing MeCP2’s LLPS capacity are associated with Rett syndrome, impacting chromosome integrity and transcriptional silence (24–26). Conversely, excessive LLPS leads to the formation of overly stable or large biomolecular condensates (BMCs), trapping essential cellular components and disrupting normal cellular processes. In neurodegenerative diseases such as ALS and frontotemporal dementia, proteins like TDP-43 and FUS undergo excessive LLPS, forming cytotoxic aggregates (27). Similarly, in Ewing sarcoma, the EWS-FLI1 fusion protein utilizes LLPS to form super-enhancers that drive oncogenic gene expression (28–32). Abnormal phase transitions within phase-separated compartments indicate pathological LLPS, transitioning from a dynamic, liquid-like state to a more solid, gel-like consistency (33). These transitions, characterized by irreversible hydrogel formation and involving proteins like FUS and SOD1, are particularly prominent in neurodegenerative disorders such as Alzheimer’s disease (33–37). These changes disrupt cellular functions by impeding molecular mobility, affecting proteostasis, and activating stress responses, thereby highlighting the extensive implications of LLPS dysregulation in disease contexts (37).

## 2 Potential roles of LLPS in respiratory diseases

Respiratory diseases cover a wide spectrum of pathologies affecting the airways, lungs, pleura, and related clinical syndromes. Clinical presentations vary widely, ranging from slight symptoms such as cough, chest pain, and mild dyspnea, to severe manifestations including respiratory distress, hypoxia, and potentially fatal respiratory failure (38). These diseases are broadly categorized based on lesion location and characteristics into obstructive, restrictive, pulmonary vascular, infectious lung diseases, and malignancies (38). Factors such as global air pollution, smoking, and aging populations significantly increase the incidence and mortality rates of chronic obstructive pulmonary disease, asthma, lung cancer, interstitial lung fibrosis, and pulmonary infections worldwide (39). Respiratory diseases have emerged as major causes of global morbidity and mortality (40). Despite this, substantial knowledge gaps persist in understanding the mechanisms and treatments of these diseases, highlighting the need for urgent and comprehensive research efforts. LLPS encompasses diverse physiological functions and is associated with a wide range of biological activities. Aberrant LLPS is implicated in multiple diseases, indicating that LLPS-induced aberrant biological activities may contribute to the pathogenesis of respiratory diseases (41). Recent studies have demonstrated that LLPS is involved in various respiratory diseases, including lung cancer, viral or fungal infections, as well as lung injury (42, 43).

### 2.1 LLPS in lung cancer

Lung cancer, characterized by uncontrolled proliferation and growth of cells, remains the leading cause of cancer-related mortality globally, accounting for approximately 18% of all cancer deaths. The etiology and pathogenesis of lung cancer are complex, involving mutations in oncogenes, inactivation of tumor suppressor proteins, and dysregulation of signaling pathways (44). Recent advancements in elucidating the molecular mechanisms underlying lung cancer have underscored the critical role of LLPS in tumorigenesis (42) (**Table 1**). Biomolecules undergo phase transitions to form membrane-less organelles or condensates. These condensates influence various cellular processes, and their dysregulation is increasingly recognized as a crucial factor in lung cancer development.

**Table 1:** Main mechanisms of LLPS in lung cancer.

Cancer	Protein or RNA	Mechanism	Reference
**NSCLC**	**EML4–ALK**	EML4-ALK condensate hyperactivates the oncogenic signaling	(45)
**Lung adenocarcinoma**	**CCDC6-RET**	CCDC6-RET condensate increases RAS signaling and MAPK signaling	(46)
**Lung cancer**	**KAT8**	KAT8 undergoes phase separation and forms a condensate with IRF1, which enhances PD-L1 expression and promotes tumor immune evasion	(47)
**NSCLC**	**YAP/TAZ**	Undergo LLPS and enhance oncogenic gene transcription	(48, 49)
**NSCLC**	**p53**	p53 mutants undergo phase separation and lead to inactivation of p53 Disruption of 53BP1 phase separation impairs p53 enrichment and compromises genomic stability	(50–52)
**NSCLC**	**MELTF-AS1**	MELTF-AS1 could directly bind and drive the phase separation of YBX1, activating ANXA8 transcription and promoting tumorigenesis of NSCLC	(53)
**Lung cancer**	**EZH2/STAT3**	Myristoylation-mediated LLPS of EZH2 compartmentalizes its non-canonical substrate, STAT3, and activates STAT3 signaling, accelerates lung cancer cell growth	(54)
**Lung cancer**	**MNX1-AS**	MNX1-AS1 promotes phase separation of IGF2BP1 to drive c-Myc–Mediated cell cycle progression and proliferation in lung cancer	(55)

**Figure 3:**
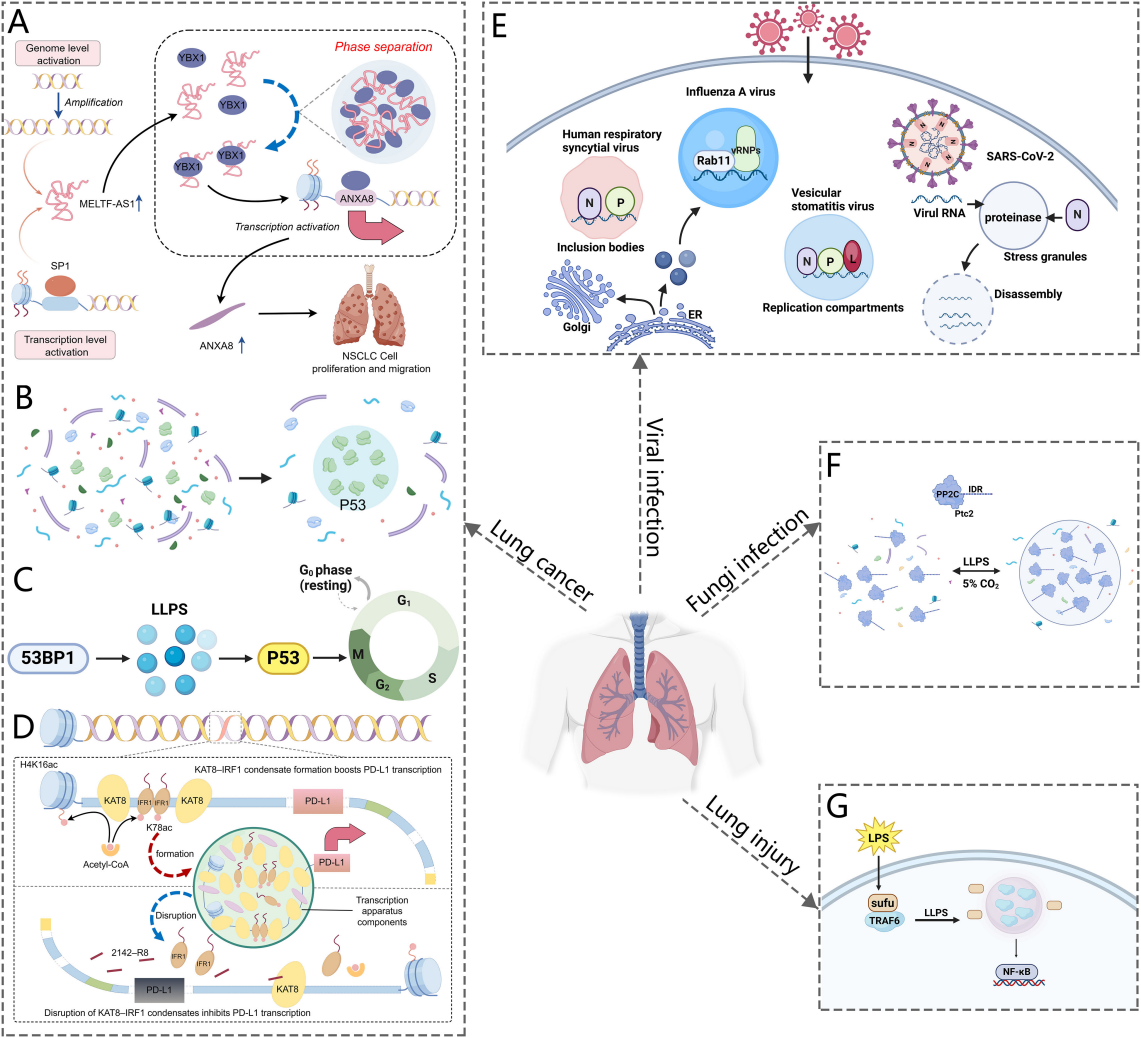
Schematic representation of LLPS in respiratory diseases. **(A)** MELTF-AS1 directly binds to YBX1, inducing its phase separation, which activates ANXA8 transcription and promotes NSCLC tumorigenesis. **(B)** P53 forms phase-separated condensates. **(C)** Phase-separated 53BP1 regulates p53 activity and maintains genomic stability. **(D)** KAT8 acetylates IRF1, forming condensates with PD-L1 and transcriptional machinery to enhance PD-L1 expression and facilitate immune evasion by cancer cells. **(E)** The SARS-CoV-2 N protein undergoes phase separation to form droplets. It interacts with G3BP1, leading to stress granule disassembly. Additionally, the virus forms liquid compartments through LLPS, which aids in viral replication and packaging. **(F)** CO_2_ induces the formation of phase-separated droplets of PP2Cs in *C albicans*. **(G)** Sufu inhibits TRAF6 droplet formation through phase separation, preventing NF-κB activation in response to LPS stimulation.

To begin with, lung cancer frequently involves mutations in oncogenic genes that are crucial for both initiating and advancing the disease. Notably, Anaplastic Lymphoma Kinase (ALK) fusions, particularly the EML4-ALK fusion, are integral to the oncogenic landscape of specific non-small cell lung cancer (NSCLC) (56–58). Research by Qin et al. has demonstrated that EML4-ALK variant 1 can form liquid-like condensates in the cytoplasm. The EML4 region of the fusion protein is crucial for this process. The EML4-N fragment alone can induce phase separation, in contrast to the dispersed nature of the ALK-C fragment (45). Mutations in aromatic residues within the EML4 region significantly disrupt phase separation, underscoring the importance of these residues. Similarly, rearranged during transfection (RET) is another important driver gene for NSCLC (59). The Coiled-coil domain containing 6 (CCDC6)-RET fusion gene undergoes LLPS in the cytoplasm independently of ligands or associated proteins (46). Studies of CCDC6-RET LLPS have revealed the simultaneous recruitment of GRB2 and SHC1, thereby establishing a membrane-less signaling microdomain that facilitates Ras/MAPK signaling transduction. Additionally, long non-coding RNAs (lncRNAs) play a crucial role in lung cancer, especially in the formation of macromolecular condensates. The oncogenic lncRNA MELTF-AS1 is upregulated in NSCLC and correlates with advanced TNM stages, larger tumor size, and poorer survival rates (53). MELTF-AS1 directly interacts with YBX1, an RNA-binding protein involved in tumorigenesis, promoting its phase separation and consequently activating ANXA8 transcription, which drives NSCLC progression (**Figure 3A**). Another lncRNA MNX1 antisense RNA 1 (MNX1-AS1) is also upregulated in NSCLC due to copy-number gain and c-Myc-mediated transcriptional activation, and is associated with poor clinical outcomes. MNX1-AS1 induces phase separation of IGF2BP1, enhancing its interaction with the 3’- untranslated region of c-Myc and E2F1 mRNAs, thereby stabilizing these mRNAs and accelerating cell-cycle progression and proliferation in NSCLC (55).

In addition to mutations in oncogenic genes, the pathogenesis of lung cancer is intricately associated with defects in tumor suppressors. p53 is a pivotal tumor suppressor essential for inhibiting cancer development. Mutations in p53 can result in the loss of its tumor-suppressive function, thereby promoting cell proliferation and inhibiting apoptosis in cancer cells (60). In lung cancer tissues, mutant p53 protein is significantly overexpressed, which correlates with a poorer prognosis in patients harboring p53 mutations (61). Recent reports indicate that p53 can also perform its functions by participating in the formation of liquid-like condensates. Firstly, p53 contributes to the formation of PML and Cajal bodies under stress response conditions (62, 63). Furthermore, p53 undergoes LLPS in response to DNA damage, forming liquid droplets that recruit and activate downstream effectors of the DNA damage response (**Figure 3B**). However, mutations in p53 can disrupt the formation of these droplets, resulting in impaired DNA repair and increased genomic instability (64). Studies have also identified the amyloid formation of p53 protein in human cancer tissues. Cancer-associated mutations in p53 accelerate protein aggregation and amyloid formation by disrupting the folding of the p53 core domain (50). Mutant p53 tends to form aggregates with amyloid properties in H1299 cells, especially amyloid oligomers inside the nucleus, which are believed to cause oncogenic gain-of-function. The pathway of mutant p53 from liquid droplets to gel-like and solid-like (amyloid) species may be a suitable target for anticancer therapy (65). Additionally, p53-binding protein 1 (53BP1), a crucial mediator of the DNA damage response and an interactor with p53, recruits other DNA repair proteins, drives transcription of p53 target genes, and affects cellular oncogenicity. Research has shown that 53BP1 undergoes phase separation to form droplet-like compartments near DNA breaks (**Figure 3C**) (52, 66). The phase-separated 53BP1 serves as a scaffold for recruiting p53 molecules and activating factors, thereby amplifying signaling and maintaining genomic integrity. Disruption of 53BP1 phase separation impairs p53 enrichment and compromises genomic stability.

Last but not least, abnormal activation of signaling pathways in lung cancer promotes the proliferation, survival, and metastasis of cancer cells. As previously discussed, LLPS serves as a pivotal hub in regulating signal transduction and modulating cellular activities. Aberrant LLPS, which disrupts signaling pathways can drive tumorigenesis and cancer progression. Dysregulation of the Hippo signaling pathway, particularly through the activation of its downstream effectors, Yes-associated protein (YAP) and transcriptional coactivator with PDZ-binding motif (TAZ), is a critical factor in the development and progression of NSCLC (67). Recent studies have shown that both YAP and TAZ undergo LLPS, forming condensates that enhance gene transcription by interacting with super enhancers (48, 49). The nuclear factor NONO promotes TAZ LLPS and facilitates its role in oncogenic transcription. Additionally, YAP undergoes LLPS to form nuclear condensates under hyperosmotic stress, which compartmentalize factors like TEAD1 and TAZ, thereby enhancing YAP-specific gene transcription (68). The small nucleolar RNA host gene 9 (SNHG9) drives LATS1 LLPS and inhibits YAP phosphorylation by LATS1 (69–71). This complex interplay highlights the potential of targeting YAP/TAZ in NSCLC, although research in this area remains ongoing. Furthermore, enhancer of zeste homolog 2 (EZH2) binds to and methylates STAT3, leading to STAT3 signaling activity by increased tyrosine phosphorylation of STAT3, and is a significant target for anticancer therapies. Recent studies have shown that myristoylation enables EZH2 to form phase-separated droplets *in vitro* and liquid-like nuclear puncta in lung cancer cells. STAT3 is observed to co-localize with EZH2 in these puncta. The interaction between EZH2 and STAT3 is further amplified by EZH2 myristoylation, leading to the activation of STAT3 signaling and the promotion of lung cancer cell growth (54). These findings provide a rationale for targeting EZH2 myristoylation, disrupting EZH2-STAT3 interactions within the condensates, or modulating the properties of EZH2 condensates. In addition to Hippo and STAT3 signaling pathway, the programmed death-1 (PD-1)/PD-ligand (L) 1 axis is also closely related to lung cancer development. Activation of the PD-1/PD-L1 pathway contributes to tumor immune escape (72). Wu et al. (47) reported that exposure to interferon-γ (IFNγ) induces phase separation of KAT8 and IRF1 in the A549 lung cancer cells, resulting in the formation of BMCs that enhance PD-L1 expression (**Figure 3D**). By exploiting the mechanism of KAT8–IRF1 condensate formation, the study identified the 2142–R8 blocking peptide, which disrupts the assembly of these condensates, thereby inhibiting PD-L1 expression and enhancing antitumor immune responses both *in vitro* and *in vivo*. These findings underscore the crucial role of KAT8–IRF1 condensates in regulating PD-L1 and propose a competitive peptide strategy to improve antitumor immunity.

Lung cancer is characterized by significant heterogeneity and complexity. Its etiology is diverse, encompassing mutations in oncogenes such as ALK and RET, inactivation of the tumor suppressor protein p53, and dysregulation of various signaling pathways. LLPS plays a crucial role in lung cancer by influencing multiple molecules and signaling pathways. Recent advancements in understanding the biophysical properties of the cancer cell microenvironment and the mechanisms of LLPS in lung cancer have created new opportunities for innovative therapeutic strategies. LLPS-based therapies involve manipulating RNA or protein interactions that govern the formation of liquid droplets within cells, including YAP, TAZ, p53, and lncRNAs. Targeting these interactions can disrupt the formation of liquid droplets that are essential for cancer cell survival. Moreover, targeting oncogenic signaling pathways, including Hippo, STAT3, and PD-L1, has been a key focus of LLPS-based therapies. Although LLPS-based therapies are still in the early stages of development, their potential for personalized cancer treatment is considerable. With ongoing research, LLPS-based targeted therapies could emerge as a cornerstone of lung cancer treatment.

### 2.2 LLPS in viral infection

Recent research has highlighted a significant link between LLPS and various non-communicable diseases, with this connection now extended to infectious diseases, particularly viral infections (73). LLPS plays a crucial role at multiple stages of the viral lifecycle, including protein synthesis, genome assembly, virus assembly, and the processes of budding and release (**Table 2**) (74). Notably, LLPS can function as both a host defense mechanism against pathogens and a tool for pathogens to enhance their invasiveness in viral infections. This dual role illustrates its complex involvement in the dynamics of viral diseases (43).

**Table 2:** Mechanism of LLPS in viral infections.

Virus	Protein	Mechanism	Reference
**Severe Acute respiratory syndrome coronavirus 2 (SARS-CoV-2)**	N	N protein condensate recruits polymerase and RNA to promote viral RNA transcription and replication.	(75, 76)
**Influenza A virus (IAV)**	Ribonucleoproteins	IBs concentrate viral ribonucleoprotein and RNA and promote the assembly of virion.	(77, 78)
**Human respiratory syncytial virus (RSV)**	N, P	IBs compartmentalize N, P protein for RNA replication	(79–81)
**Measles virus (MeV)**	N, P	Both viral N protein and P protein trigger the formation of IBs containing the cellular protein WDR5	(82, 83)
**Vesicular stomatitis virus (VSV)**	N, P, and L	Viroplasm formation involves the assembly of a liquid compartment driven by the N, P, and L proteins	(84)
**Infectious Bronchitis Virus (IBV)**	nsp15	Endonuclease nsp15 inhibits the formation of host anti-viral SGs to ensure efficient replication	(85)
**Human adenoviruses (HAdV)**	DNA-binding protein (DBP)	DBP is a major component of replication compartments and exhibits the properties of a biomolecular condensate	(86)
**Herpes simplex virus type 1 (HSV1)**	ICP4, UL11	ICP4 regulates protein condensation and LLPS through its intrinsically disordered regions (IDRs) Membrane protein UL11 is an intrinsically disordered, conformationally dynamic protein that regulates LLPS by binding multiple chaperone proteins	(87, 88)
**Human Metapneumovirus (HMPV)**	P	IBs possess the properties of liquid organelles, and the purified P protein phase separates independently *in vitro*	(89)

The nucleocapsid (N) protein plays a crucial role in viral replication, the packaging of viral genomic RNA into new virions, and the modulation of the host cell’s response to infection. The emergence of SARS-CoV-2 has led to a global health crisis known as COVID-19, presenting significant challenges to public health systems worldwide (90). Studies have indicated that the nucleocapsid (N) protein of SARS-CoV-2 undergoes LLPS, forming condensates that are rich in RNA and polymerase (**Figure 3E**) (43, 91). The N protein comprises two globular domains: the RNA-binding domain and the C-terminal dimerization domain, flanked by intrinsically disordered regions (IDRs) (92). This structure facilitates LLPS in the presence of RNA, with the process being modulated by the concentrations of RNA and proteins (93). Phase separation generates spherical droplets that are observable under fluorescence microscopy. These droplets undergo material transformations over time, potentially indicating the initial stages of nucleocapsid assembly. Moreover, the N protein is associated with SGs, cytoplasmic structures formed through LLPS, and plays a crucial role in viral replication efficiency. In cells, the N protein colocalizes with G3BP1, a marker protein for SGs. Fluorescence recovery after photobleaching (FRAP) analysis reveals distinct populations of the N protein within SGs, suggesting diverse sub-structural localizations and functions. Researchers have identified that the N protein can be recruited into SGs, where it interferes with the interaction between G3BP1 and other core SGs components, leading to SGs disassembly (**Figure 3E**) (94). Guseva et al. (82) demonstrated that purified N protein and phosphoprotein (P) from measles virus (MeV) formed liquid-like, membrane-less organelles upon *in vitro* mixing. They identified weak interactions involving the intrinsically disordered domains of the N and P proteins, which are essential for phase separation. RNA colocalized with droplets, initiating the assembly of N protein protomers into nucleocapsid-like particles that encapsulated the RNA. The rate of encapsidation within droplets was enhanced compared to the dilute phase, revealing a significant role of LLPS in MeV replication.

During viral infection, the replication and assembly of numerous viruses take place within specialized intracellular compartments. These structures are known as viral factories, replication compartments (RCs), inclusion bodies (IBs), SGs, Negri bodies (NBs), and cytoplasmic virion assembly compartments (cVACs). These compartments concentrate viral proteins, nucleic acids, and cellular factors, facilitating the essential steps of viral replication and concurrently shielding the viral genome from cellular defenses. LLPS may contribute to the formation and maintenance of these compartments. Influenza A virus (IAV) infections represent a significant threat to human health, resulting in annual epidemics and occasional pandemics (95). Alenquer et al. demonstrated that during viral assembly, IAV forms cytosolic inclusions comprising viral ribonucleoproteins. Their study revealed that these viral inclusions possess characteristics akin to liquid organelles, segregating from the cytosol without a defining membrane, dynamically exchanging material, and rapidly adapting to environmental changes (77). Evidence was provided that viral inclusions localize near endoplasmic reticulum (ER) exit sites, rely on continuous ER-Golgi vesicular cycling, and do not provoke an interferon response (**Figure 3E**). Additionally, the study proposes that viral inclusions isolate ribonucleoproteins from the cytosol and enable specific RNA-RNA interactions within a liquid environment. The RCs of vesicular stomatitis virus (VSV) also display liquid-like properties resulting from LLPS. Analysis of the expression of individual viral components involved in replication has revealed that three key viral proteins, namely the N, P protein and the multifunctional large protein (L), are essential for replication and can induce LLPS within the cytoplasm (**Figure 3E**) (84). Human adenoviruses (HAdV) are prevalent pathogens responsible for acute respiratory tract infections and have recently shown an increased prevalence, often leading to pneumonia (96, 97). Hidalgo et al. (86) investigated the biophysical properties of intranuclear RCs formed during HAdV infection. They identified the viral DNA-binding protein (DBP) as a major component of RCs, which contains intrinsically disordered and proline-rich regions—features also found in proteins involved in phase transitions. Through FRAP and time-lapse studies in living HAdV-infected cells, they observed that DBP-positive RCs display characteristics similar to liquid BMCs, including fusion and division, eventually forming an intranuclear mesh with diminished fluid-like properties. Additionally, the transient expression of DBP replicates the assembly and liquid-like properties of RCs in HAdV-infected cells. These findings indicate that DBP may act as a scaffold protein in the assembly of HAdV-RCs, thereby guiding future research into the role of BMCs in virus-host interactions. Respiratory syncytial virus (RSV), a major cause of acute respiratory infections in young children and a significant concern for the elderly and immunocompromised, has also been studied in this context (98). Research has shown that cytoplasmic IBs, which are essential for viral replication and transcription, depend on LLPS mediated by interactions between the N and P proteins (**Figure 3E**) (74, 79, 98). In addition to RSV, Boggs et al. (89) showed that Human metapneumovirus (HMPV) IBs in infected or transfected cells display properties characteristic of liquid organelles, including fusion and fission. Purified HMPV P protein was found to form liquid droplets independently, differentiating it from IBs in other viral systems. *In vitro* experiments demonstrated that the HMPV P protein recruits monomeric N (N0-P) and N-RNA to form droplets. This observation suggests that the P protein functions as a scaffold, facilitating multivalent interactions with both monomeric and oligomeric N protein and RNA, thereby promoting phase separation of IBs. Moreover, recent studies provided evidence that the endoribonuclease nsp15 of Infectious Bronchitis Virus (IBV) interferes with the formation of host antiviral SGs by regulating the accumulation of viral dsRNA and by antagonizing the activation of protein kinase R, eventually ensuring productive virus replication.

LLPS is also critical for herpesvirus replication and transcription. For example, the herpes simplex virus type 1 (HSV-1) immediate-early protein ICP4, which is intrinsically disordered, induces the formation of nuclear condensates via LLPS (73). The role of ICP4 in viral replication, along with its localization within replication compartments, suggests that LLPS may contribute to the formation of these compartments (88, 99). Additionally, HSV-1 tegument protein UL11, which contains IDRs, also undergoes LLPS *in vitro* (87, 100). The presence of IDRs in several HSV-1 tegument proteins suggests a potential role in tegument assembly through LLPS (87).

The significance of LLPS in viral processes associated with respiratory infections, such as adsorption, replication, assembly, and release, has become increasingly clear (73, 74). LLPS promotes the formation of BMCs, which concentrate viral replication machinery, enhance viral gene transcription and expression, and modulate innate immune responses by sequestering host sensors within IBs (43). This mechanistic insight into phase separation provides a deeper understanding of the viral life cycle within host cells and suggests potential strategies for developing targeted treatments for viral infections (101).

### 2.3 LLPS in fungi infection

*Candida albicans*, commonly known as a yeast species, typically has a symbiotic relationship with the human body under normal conditions, but in cases of weakened immune systems, it can proliferate uncontrollably, leading to candidiasis pneumonia (102). This condition is often characterized by respiratory distress, coughing, chest pain, and fever (103). Zhang et al. (104) discovered that elevated CO2 levels induce the formation of IDR-containing PP2Cs in *Candida albicans*. The Ser/Thr-rich sequences within the IDRs are essential for driving the phase transitions of PP2Cs under high CO2 conditions. Their findings suggest that interactions between Ser/Thr residues and CO2 may enhance the cohesive forces required for phase separation. This phase separation activates the phosphatase activity of PP2Cs, thereby initiating various CO2-responsive biological processes (**Figure 3F**). Their data demonstrated that the functionally conserved, yet sequence-diverse, disordered regions of PP2C phosphatases can act as CO2 sensors.

### 2.4 LLPS in lung injury

Sepsis is marked by severe organ dysfunction, especially in the lungs, due to a dysregulated immune response to infections (103). Recent studies have shown decreased expression of Suppressor of Fused (Sufu) during the early stages of lipopolysaccharide (LPS)-induced acute inflammation in murine lung and peritoneal macrophages. The absence of Sufu worsens lung injury and increases mortality in mice subjected to LPS and cecal ligation and puncture (CLP) challenges (105). Moreover, Sufu deficiency amplifies LPS-induced proinflammatory gene expression in cultured macrophages (105). This research underscores Sufu’s critical role as a negative regulator in the Toll-Like Receptor (TLR)-mediated inflammatory response. Sufu directly interacts with TNF Receptor Associated Factor 6 (TRAF6), inhibiting its oligomerization and autoubiquitination. During LPS-induced inflammation, TRAF6 undergoes phase separation, a crucial step in the activation of ubiquitination and NF-κB signaling (**Figure 3G**). Sufu effectively prevents the formation of phase-separated TRAF6 droplets, thereby suppressing NF-κB activation in response to LPS. In a septic shock model, TRAF6 depletion alleviated the exacerbated inflammatory phenotype in mice with myeloid cell-specific Sufu deletion.

## 3 LLPS as a novel therapeutic mechanism

**Figure 4:**
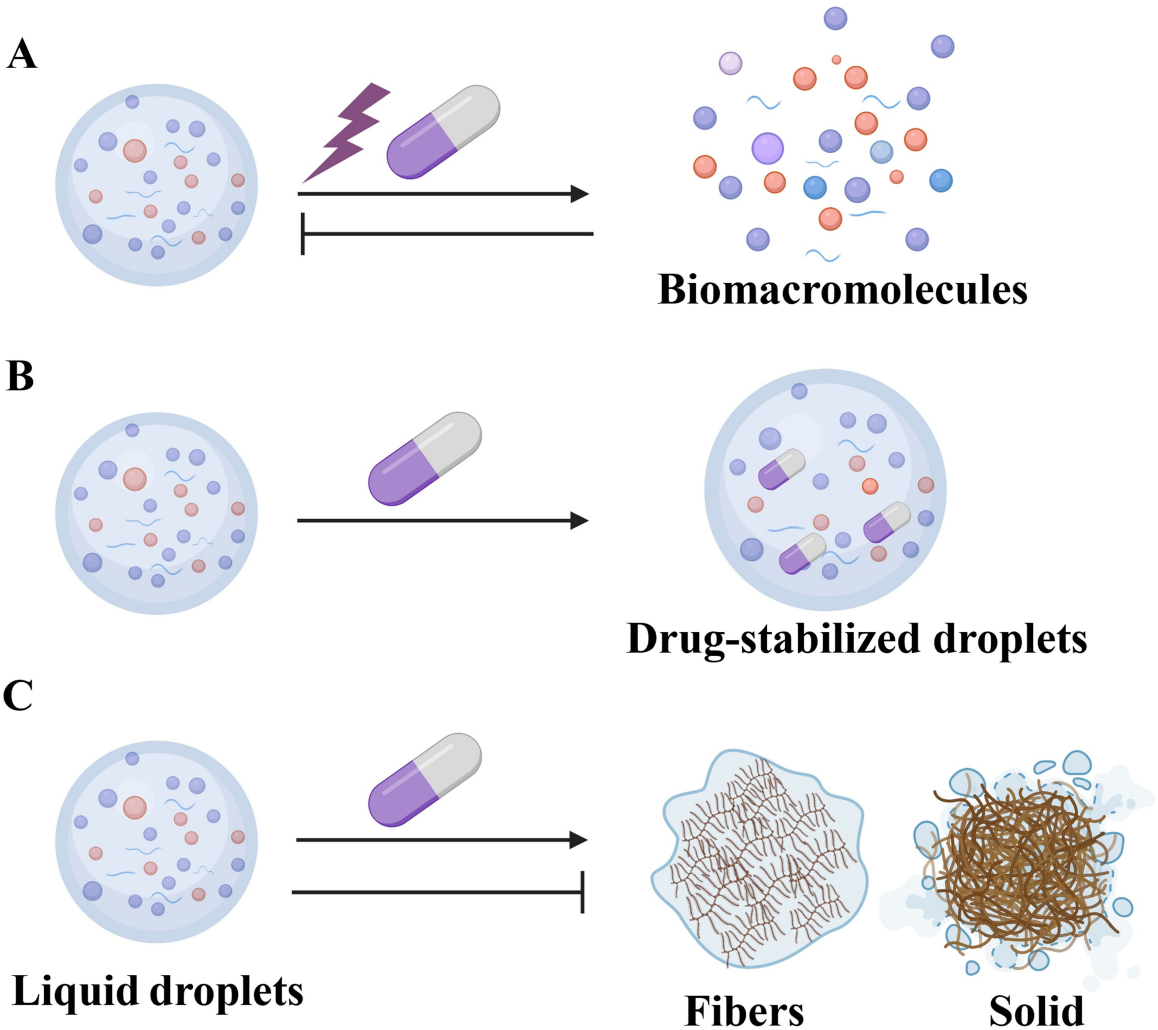
Therapies Targeting LLPS. **(A)** Drugs such as GO289, 1,6-hexanediol, GCG, A3E, and EVG, as well as environmental adjustments, can disrupt or prevent the formation of specific biomacromolecules into droplets. **(B)** Drugs, including cisplatin, can accumulate within condensates. **(C)** The steroidal alkaloid cyclopamine and A3E can solidify condensates. Conversely, other drugs may stabilize these liquid droplets, thus preventing their dissolution or transition into fibrous or solid phases.

The significance of LLPS in respiratory diseases highlights its potential as a therapeutic target. Indeed, various drugs or molecules can modify the formation rate, composition, stability, and physical properties of LLPS, thus presenting significant opportunities for drug discovery (**Figure 4**). As previously mentioned, LLPS plays a crucial role in viral infections, indicating that targeting the phase separation process could pave the way for new antiviral drug development. he SARS-CoV-2 N protein interacts with SGs proteins G3BP1/2 and CSNK2B/CSNK2A2, which are subunits of casein kinase 2 (CK2), leading to the disassembly or inhibition of SGs formation. Consequently, CK2 inhibitors such as GO289 and silmitasertib, which disrupt protein-protein interactions between the N protein and SG-associated proteins, could serve as potential chemical probes or antivirals targeting SGs (**Figure 4A**) (106, 107). Additionally, NF-κB represents a potential therapeutic target for infectious diseases. The compound 1,6-hexanediol, known to inhibit LLPS, suppresses N protein phase separation, thus limiting its role in NF-κB activation (**Figure 4A**) (108, 109). However, 1,6-hexanediol exhibits high cytotoxicity and inhibits phosphatase and kinase activities. Propylene glycol, in contrast, offers a non-toxic alternative for disrupting viral replication (110) (–).-Gallocatechin gallate (GCG), a green tea polyphenol, disrupts N protein LLPS and inhibits SARS-CoV-2 replication, potentially treating COVID-19 by targeting N-RNA condensation (**Figure 4A**) (91). Recent studies have shown that the aminoglycoside kanamycin disrupts LLPS in SARS-CoV-2-infected mammalian cells (93). Kanamycin binds to nucleic acids through electrostatic interactions, thereby preventing RNA-protein interactions. The addition of kanamycin to droplets resulted in a reduction in condensate size, a decreased protein/RNA ratio in assays, and the relocalization of the N protein to the nucleus (93). Small-molecule modulators of host kinases or phosphatases could regulate LLPS and act as antiviral agents. Activators of SR protein kinase 1 (SRPK1) are potential antivirals, as they phosphorylate the N protein SR region to attenuate RNA-induced LLPS and viral RNA transcription (111). RSV replication occurs within IBs. The steroidal alkaloid cyclopamine and its chemical analogue A3E inhibit RSV replication by disrupting and solidifying IB condensates (**Figures 4A, C**) (98). Furthermore, cyclopamine and A3E demonstrate dose-dependent inhibition of RSV replication in mouse models, highlighting their therapeutic potential.

In addition to direct interventions targeting LLPS formation, modifying environmental conditions can regulate the LLPS states of target proteins. SGs are particularly sensitive to oxidative, osmotic, and heat shock stresses. Temperature variations may thus affect translation mechanisms within SGs under stress (112). Furthermore, SARS-CoV-2 N protein condenses with specific RNA genomic elements under physiological buffer conditions and condensation is enhanced at human body temperatures (33°C and 37°C) and reduced at room temperature (22°C) (113). Roden et al. also observed that N protein can condense in the absence of RNA at high temperatures, but addition of RNA lowers the temperature at which droplets emerge (114). These findings highlight the potential of environmental modulation, such as temperature adjustment, as a strategy to disrupt LLPS and inhibit viral replication, suggesting it could be a promising adjunctive systemic therapy (**Figure 4A**).

As our understanding of the role of LLPS in various biological processes advances, its potential to modulate tumor-associated proteins and their associated upstream and downstream signaling pathways is increasingly recognized, thereby improving therapeutic strategies for lung cancer. Although anti-PD-1/PD-L1 therapy has shown efficacy in lung cancer, drug resistance remains a significant challenge. Recent studies have shown that YAP interacts with histone acetyltransferase EP300, transcription factor TEAD4, and mediator 1 to form phase-separated transcriptional condensates, which enhance the transcription of target genes (115). Disrupting YAP’s LLPS capacity inhibits cancer cell growth, enhances the immune response, and increases the sensitivity of tumor cells to anti-PD-1 therapy. Another study found that SRC-1 co-expresses with YAP in NSCLC and is essential for lung cancer growth. SRC-1 interacts with YAP/TEAD to enhance YAP transcriptional activity through the formation of compartmentalized SRC-1/YAP/TEAD condensates. The anti-HIV drug elvitegravir (EVG) specifically disrupts SRC-1 condensate formation in cells, thereby efficiently inhibiting YAP oncogenic transcriptional activity and constraining YAP-dependent cancer cell growth (**Figure 4A**) (116).

Recent studies indicate that antitumor drugs accumulate in specific condensates within cancer cells, with their distribution profoundly affecting drug efficacy (**Figure 4B**). Furthermore, modifications to the properties of phase-separation condensates can further influence drug concentrations and effectiveness (**Figure 4C**). Cisplatin is a potent chemotherapeutic agent used to treat lung cancer through its ability to induce DNA damage and cell apoptosis. Studies have demonstrated that, *in vitro*, reconstituted condensates can concentrate various anticancer drugs, including cisplatin, mitoxantrone, tamoxifen, THZ1, and JQ1, all of which interact with DNA (117). These drugs showed high enrichment within the condensates of the transcription regulator MED1, which are formed by super-enhancers and large enhancer clusters associated with oncogenesis and gene activation (117, 118). Co-incubation of DNA with cisplatin in reconstituted MED1 condensates led to substantial DNA modification through platination (119). In cancer cells, plastinated DNA frequently co-localizes with MED1 condensates, suggesting that the partitioning of cisplatin into these condensates enhances drug activity (117, 119). Notably, reconstituted MED1 condensates gradually dissolved in the presence of cisplatin, and prolonged cisplatin exposure led to MED1 depletion from super-enhancers in cancer cells (117, 119). These findings suggest that the selective partitioning and concentration of antineoplastic drugs within condensates play a critical role in drug pharmacodynamics. Further investigation of this phenomenon could advance therapeutic approaches for diseases.

Modulating the assembly and dynamics of LLPS condensates enables precise control over cellular processes. This approach offers a unique opportunity to restore normal cellular functions and mitigate disease progression. LLPS-based therapies have shown considerable promise in treating respiratory diseases. However, several challenges must be addressed before these therapies can be transitioned into clinical practice. A major obstacle is the limited understanding of the specific mechanisms of LLPS in respiratory diseases and the complex signaling pathways involved. Another significant challenge involves developing efficient and precise LLPS inhibitors, as well as evaluating their safety and toxicity. Moreover, most studies have been conducted from a monocellular perspective, limiting observations to narrow contexts and overlooking complex intercellular interactions and the intricate body system. Transitioning from *in vitro* to *in vivo* models, as well as from cellular to animal models, is crucial for elucidating the role of phase separation in biochemical reactions. This advancement could significantly enhance drug targeting strategies that focus on phase separation. Addressing these challenges requires future research to further unravel the mechanisms of LLPS in respiratory diseases and identify novel LLPS regulators and therapeutic targets.

## 4 Perspectives

LLPS orchestrates the formation of various biomacromolecular condensates, which are crucial for numerous cellular processes, such as subcellular compartmentalization, cell cycle regulation, signal transduction, gene expression modulation, and protein quality control. Dysregulated phase separation, resulting from mutations in phase-separated proteins, compromised quality control systems, or environmental changes, has been implicated in the pathogenesis of various respiratory diseases. Consequently, identifying molecules that modulate phase separation represents a promising strategy for drug development and therapeutic interventions targeting diseases associated with abnormal LLPS.

Interest in the role of LLPS in respiratory diseases, particularly lung cancer and respiratory viral infections, is growing, as abnormal LLPS emerges as a crucial pathophysiological factor. Research into therapeutic strategies and medications targeting LLPS in these diseases is also expanding. Nevertheless, research connecting LLPS to lung injury and fungal infections remains limited and warrants further investigation. Although direct evidence linking LLPS to other respiratory diseases, such as chronic obstructive pulmonary disease (COPD), pulmonary fibrosis, and asthma, is scarce, emerging data suggest plausible associations. For instance, lncRNAs, functioning as architectural RNAs, recruit proteins that influence disease progression in the respiratory system. Studies have confirmed that certain lncRNAs are involved in regulating LLPS (120, 121). Future research should explore how LLPS interacts with these lncRNAs during the progression of respiratory disease. Aberrant protein expression, misfolding, and functional dysregulation are implicated in the initiation and progression of respiratory diseases. Recent studies have identified many proteins capable of LLPS, including N protein and p53, which are involved in disease pathogenesis (37, 122). Databases such as DrLLPS and LLPSDB compile extensive datasets of over 400,000 known and potential LLPS-related proteins, thereby providing crucial support for advancing LLPS research (123, 124). Investigating the ultrastructure and functional consequences of previously unknown proteins through these databases is of significant value. Further research will elucidate the precise roles of additional abnormal LLPS proteins in the pathogenesis of respiratory diseases. Additionally, SGs are membrane-less organelles formed through LLPS in response to various stress stimuli, have been identified in diseases such as asthma, COPD, and lung cancer (125).

Future research should prioritize the development and optimization of LLPS-modulating agents, focusing on their safety and efficacy in clinical settings. Exploring the broader implications of LLPS in various respiratory diseases, as well as combinatorial therapies involving LLPS modulators, may lead to innovative treatments. A thorough understanding of LLPS mechanisms in pathogenesis will be essential for designing targeted interventions. Additionally, modulating the environment, particularly through temperature control, shows promise for disrupting LLPS and potentially inhibiting viral replication, thereby complementing other therapeutic strategies. In oncology, targeting LLPS mechanisms in cancer cells, particularly those involving tumor-associated proteins and signaling pathways, may enhance therapeutic strategies, address drug resistance, and improve outcomes for patients with lung cancer. The accumulation and partitioning of antitumor drugs within specific cellular condensates offer a promising avenue for refining cancer therapy. Advancing our knowledge in these areas will enable the development of more effective treatment strategies and the optimization of therapeutic outcomes. Addressing the challenges related to LLPS mechanisms and translating findings from monocellular models to *in vivo* systems will be crucial for advancing LLPS-based therapies. Continued research into LLPS holds the potential to significantly impact the treatment of both respiratory diseases and lung cancer.

Overall, research into the relationship between respiratory diseases and liquid-phase separation is still in its early stages but is beginning to demonstrate significant potential. As technology and research advance, this field is anticipated to offer valuable insights and methods for the early diagnosis and personalized treatment of respiratory diseases.
